# Predicting the evolution of neck pain episodes in routine clinical practice

**DOI:** 10.1186/s12891-019-2962-9

**Published:** 2019-12-26

**Authors:** Francisco M. Kovacs, Jesús Seco-Calvo, Borja M. Fernández-Félix, Javier Zamora, Ana Royuela, Alfonso Muriel

**Affiliations:** 1 Unidad de la Espalda Kovacs, Hospital Universitario HLA-Moncloa. University Hospital, Avenida de Menéndez Pelayo, 67, 28009 Madrid, Spain; 2 Spanish Back Pain Research Network, Madrid, Spain; 3 Institute of Biomedicine (IBIOMED), University of León, Spain, León, Spain; 4 University of the Basque Country, Spain.CampusUniversitario, 24071 León, Spain; 5 CIBER Epidemiología y Salud Pública (CIBERESP), Madrid, Spain; 6 Unidad de Bioestadística Clínica, Hospital Ramón y Cajal. IRICYS, Madrid, Spain; 7 Barts and The London School of Medicine and Dentistry, Queen Mary University of London, London, UK; 8 Clinical Biostatistics Unit, Puerta de Hierro University Hospital, Instituto de Investigación Puerta de Hierro (IDIPHISA), Madrid, Spain

## Abstract

**Background:**

The objective of this study was to develop models for predicting the evolution of a neck pain (NP) episode.

**Methods:**

Three thousand two hundred twenty-five acute and chronic patients seeking care for NP, were recruited consecutively in 47 health care centers. Data on 37 variables were gathered, including gender, age, employment status, duration of pain, intensity of NP and pain referred down to the arm (AP), disability, history of neck surgery, diagnostic procedures undertaken, imaging findings, clinical diagnosis, and treatments used. Three separate multivariable logistic regression models were developed for predicting a clinically relevant improvement in NP, AP and disability at 3 months.

**Results:**

Three thousand one (93.5%%) patients attended follow-up. For all the models calibration was good. The area under the ROC curve was ≥0.717 for pain and 0.664 for disability. Factors associated with a better prognosis were: a) For all the outcomes: pain being acute (vs. chronic) and having received neuro-reflexotherapy. b) For NP: nonspecific pain (vs. pain caused by disc herniation or spinal stenosis), no signs of disc degeneration on imaging, staying at work, and being female. c) For AP: nonspecific NP and no signs of disc degeneration on imaging. d) For disability: staying at work and no signs of facet joint degeneration on imaging.

**Conclusions:**

A prospective registry can be used for developing valid predictive models to quantify the odds that a given patient with NP will experience a clinically relevant improvement.

## Background

Neck pain (NP) is defined as pain in the neck with or without pain referred into one or both upper limbs (“arm pain” -AP-). Worldwide, the point prevalence of NP is 4.9% (95% CI 4.6 to 5.3), and it is one of the top five chronic pain conditions in terms of prevalence and years lost to disability [1–3].

Most episodes of acute NP improve spontaneously, but more than 30% of patients show some persistent or recurrent symptoms 1 year later [3]. Early identification of patients at higher risk of pain becoming chronic, would help select those in whom more aggressive treatments are worth considering. Therefore, the development of prediction rules in this field has been recommended as a research priority [4]. Unfortunately, many of the existing prediction rules derive from studies with small samples, or from re-analyses of data gathered in experimental studies, which may produce results that differ from those in routine practice [5].

Data from registries implemented in routine practice could be useful to develop prediction rules factoring in each patient’s personal and clinical characteristics, including treatments received. This would also empower patients for informed shared decision making [5].

Ideally, registries should include virtually all the patients, gather valid, reliable and clinically relevant data, and ensure that losses to follow-up are kept to a minimum. Concerns have been expressed with regard to the feasibility of these requirements in routine clinical practice [6].

Therefore, the objectives of this study were; a) to explore the feasibility of implementing a registry of patients treated for NP in routine practice, b) use it to develop predictive models in order to quantify the likelihood that a given patient experiences a clinically relevant improvement in NP, arm pain and disability, and c) to assess such predictive models.

## Methods

### Setting

Forty-seven health care centers were selected by the Spanish Back Pain Research Network to be invited to participate in this study, based on their past involvement in research on neck and low back pain. The centers were located across 11 out of the 17 Administrative regions in the country (Andalucía, Aragón, Asturias, Baleares, Castilla-León, Cataluña, Extremadura, Galicia, Madrid, Murcia, Vascongadas). The population of these regions is 35,776,167, approximately 77% of the total population of the country [7].

Fifteen centers belonged to the Spanish National Health Service (SNHS), 6 to not-for-profit institutions working for the SNHS, and 26 were private. They included 8 primary care centers, 18 physical therapy practices, and 21 specialty Services in rheumatology (5), rehabilitation (6), neuroreflexotherapy (4), and orthopedic surgery (6).

### Subjects

The inclusion and exclusion criteria were as follows.

Inclusion criteria: a) Seeking care for NP in a participating unit, b) suffering from neck pain, with or without pain in the arm, unrelated to trauma or systemic disease, and c) proficiency in Spanish.

Pain unrelated to systemic disease was defined as pain unrelated to cancer or inflammatory disease (e.g., rheumatoid arthritis), in patients who did not present signs suggesting fibromyalgia (defined as diffuse pain with unexplained fatigue or sleep disturbances) or “red flags” for latent systemic diseases. “Red flags” were defined as “oncologic disease during the previous 5 years, constitutional symptoms –unexplained weight loss, fever, chills-, history of intravenous drug use, or immunocompromised host” [8–11]. Patients with “red flags” could be included in the study if the appropriate diagnostic test had ruled out the presence of systemic diseases.

Patients who were included were asked to sign an informed consent, authorizing the use of demographic and clinical data related to their care for the purpose of this study.

Exclusion criteria were: central nervous system disorders (treated or untreated), other causes of referred or radiated pain in the arm (e.g., peripheral nerve damage) and not having signed the informed consent.

In order to analyze the influence of up to 40 variables, the sample had to include at least 400 subjects who would not experience improvement [12]. Approximately 80–85% of patients with spinal pain, experience a clinically relevant improvement in pain, referred pain and disability, at 3 months, while losses to follow-up at that period range between 5 and 10% [13–16]. Therefore, sample size was established at 2934 subjects. There were no concerns about the sample size being too large, due to the observational nature of the study.

### Procedure

Since this study did not require any changes to standard clinical practice, according to the Spanish law it was not subject to approval by an Institutional Review Board. All procedures followed were in accordance with the ethical standards of the Helsinki Declaration of 1975, as revised in 1983.

As per standard practice in Spain, patients and clinicians received no compensation for participating in this study.

Patients were recruited consecutively at the participating centers. All patients seeking care for NP were screened for inclusion and exclusion criteria.

All patients complying with inclusion criteria were invited to participate, and all those who accepted to sign the informed consent were included. Recruiting clinicians explained to eligible patients the importance of answering fully and accurately a series of questionnaires assessing their clinical status, and complying with the follow-up visit for an assessment of their evolution.

Patients were assessed upon recruitment and at follow-up. The follow-up assessment was planned at 3 months because: a) this study sought to analyze the outcome of a single episode of neck pain rather than relapses, b) this timeframe implies that all patients who are symptomatic at follow-up, would be chronic [17]; c) existing studies conducted in the environment where this study took place, have shown that losses to follow-up remain minimal for periods of up to 3 months [15, 18, 19], rise at 6 months [20–22], and become increasingly significant thereafter [13, 23].

Patients were asked to complete the self-administered questionnaires at both assessments. Questionnaires were completed in private, with no interference from health care personnel or any other actors. Data from the questionnaires were inserted into a database by a team of auxiliary personnel with no connection to the treating physician. In order to make clinical decisions, the treating clinicians had access to that information, but were not able to alter the data. Participating in this study did not imply any changes to patients’ clinical management, and clinicians were instructed to manage their patients as usual.

### Variables

This registry used the same variables and measuring instruments as the only registry of neck and back pain patients available in Spain. The latter was originally developed for post-marketing surveillance of a minimally invasive technology (“neuro-reflexotherapy”), and has shown to be reliable and lead to low proportions of missing data and losses to follow-up [14, 16, 24].

The registry gathered data from patients and from clinicians. Data requested from patients at the first assessment, were: gender, age (date of birth), duration of the current pain episode (date of pain appearance), time elapsed since the first episode (years), and employment status (classified as working, on sick leave, receiving disability compensation, student, housewife, unemployed, retired, or other; at the analysis phase, these categories were collapsed into: “working”, “receiving financial compensation for NP” -on sick leave or disabled for that reason-, or “non worker” –any other status-).

At both assessments, patients were asked to report pain and disability, which are considered two of the main outcome measures for patients with spinal pain [25]. To this end, they completed two separate 10-cm visual analog scales for NP and AP (−VAS-, for which 0 = no pain and 10 = worst imaginable pain) [26], and a validated Spanish version of the Neck Disability Index (−NDI-, for which 0 = no disability and 100 = worst possible disability) [18].

Data requested from recruiting clinicians were: diagnostic procedures prescribed for the current episode (X-Rays, CT scan, MRI, EMG, other -blood analyses, scintigraphy, etc.-), patients’ radiological findings on imaging procedures performed for the current or previous episodes, as reported by radiologists (disc degeneration, facet joint degeneration, scoliosis, difference in leg length, spondylolisis, spinal stenosis, annular tear, disc protrusion, disc herniation, other radiological findings, no findings), diagnosis (pain caused by disc herniation, spinal stenosis or “common non-specific NP”), and treatments undergone by the patient throughout the study (drugs –analgesics, NSAIDs, steroids, muscle relaxants, opioids, other drugs-, physiotherapy and rehabilitation -which were collapsed into a single category at the analysis phase, and were defined as any form of exercise, heat, cold, electrotherapy or hands-on techniques, such as massage or mobilization-, neuroreflexotherapy intervention -defined as the implantation of surgical material in specific areas of the skin, for up to 90 days- [27], surgery, other treatments -e.g., spinal injections-).

Pain caused by disc herniation or spinal stenosis, was diagnosed if radicular pain or neurologic signs existed, and were consistent with the location in which disc protrusion/herniation or spinal stenosis had been documented on MRI. Patients not fitting into these definitions, were classified as suffering from “common, non-specific NP”.

### Analysis

All the analyses were undertaken by a team of independent biostatisticians who had no contact or communication with the clinicians involved in this study.

For categorical variables, absolute and relative frequencies were calculated. When distribution was normal, values for continuous variables were described through their mean and standard deviation (SD). When their distribution departed from normality, median and percentiles 25 and 75 were used.

Reductions in VAS or NDI scores between the baseline and follow-up assessments, were considered to reflect improvement only if they were greater than the minimal clinically important change (MCIC). The MCIC for pain and disability has been established as 30% of their baseline scores, with a minimum value of 1.5 for VAS and 7 NDI points for neck pain-related disability [19, 28]. This implied that it was impossible for patients with baseline scores below these values, to show a clinically relevant improvement at follow-up.

For instance, it was impossible for a patient with a disability score smaller than the MCIC at baseline to experience improvement in disability, and including this patient’s data into the model exploring factors associated with a clinically relevant improvement in disability could skew results. In the case of patients with baseline scores below the MCIC, lack of improvement could only be spotted if they experienced a worsening of pain or disability (i.e., an increase in the score at follow-up, as compared to baseline). Therefore, in the case of patients with a baseline score which was smaller than the MCIC for a given variable, only those who at discharge had shown to have experienced a worsening were included in the analyses.

Outcome measures were neck pain, arm pain and disability. In order to quantify the likelihood that a given patient would experience a clinically relevant improvement in these variables, three separate multivariable predictive logistic regression models were developed. Improvement in NP, AP or disability were the dependent variables, and the maximal models included: gender; age (in years); baseline intensity of NP (VAS points); baseline intensity of AP (VAS points); NP-related disability at baseline (NDI percentage); duration of the current episode in days (number of days); duration of the current episode (classified as acute or chronic, with a cut-off limit at 90 days) [17]; time elapsed since the first episode (years), employment status (“working”, which was the reference category, “non worker” or “receiving financial compensation for NP”); diagnostic tests undertaken at any moment during the study period (X-Rays, MRI, other); findings in imaging procedures undertaken during the study period or previous episodes (disc degeneration, facet joint degeneration, scoliosis, spondylolisis, spinal stenosis, disc protrusion, disc herniation, other findings, no findings); clinical diagnosis (pain caused by disc protrusion/hernia, pain caused by spinal stenosis, or non-specific, common neck pain); and treatments used during the study period (drugs -analgesics, NSAIDs, steroids, muscle relaxants, opioids, other-, physiotherapy, rehabilitation, neuro-reflexotherapy, surgery).

The value *p* > 0.05 was used to eliminate variables from the model, following a non-automatic backward strategy. Nomograms were developed to illustrate the results of the models [12].

The area under the ROC curve (AUC) was used to asess discrimination of the final models, and calibration was assessed using the Hosmer-Lemeshow test [12].

The selection of variables was validated by using bootstrapping: 100 bootstrap samples were drawn [29]. Sample size was estimated using the number of observations that contained no missing values [29, 30]. The variables selected were displayed for each sample drawn, and the total number of times each variable was selected was counted [29].

Both the apparent performance of each bootstrap sample (“bootstrap performance”) and the performance of the bootstrap model in the original sample (“test performance”), were determined. Overfitting was defined as the average difference between bootstrap performance and test performance [31].

The Stata/IC v15.1(StataCorp, 2017) was used for statistical analysis.

## Results

All the 47 health care centers which were invited to participate in this study, accepted. All patients invited to participate in the study, agreed to sign the informed consent. All were included and none were excluded. Due to the multicenter design, when sample size was reached, 3225 patients had been included. The second assessment, which was planned 90 days after the first one, generally took place later (median [p25;p75] = 103 [89;137] days), and 224 patients (6.95%) were lost to follow up.

Among the 3001 patients who attended the follow-up, most were women (74.2%), the median duration of their pain episode was 180 days, and their baseline median value for pain intensity was 6.6 VAS points for NP and 6.0 for AP, while their mean score for disability in the NDI was 37.2 (Table 1). The baseline characteristics of patients who were lost to follow up, were similar (data not shown).

**Table 1:** Characteristics of patients recruited, diagnostic tests and treatments used during the study

Variables	*n* = 3001
Gender (female)^a^	2227 (74.2)
Age (years)^b^	50.3 (16.0)
Employment status^a^	
Non worker	1166 (38.8)
Active	1453 (48.4)
Duration of the pain episode (days) ^c^	180 (90; 365)
Duration of the pain episode categorized (days)^a^	
Acute (< 90 days)	971 (32.4)
Chronic (≥90 days)	2030 (67.6)
Time elapsed since first episode^a^	
< 1 year	648 (21.6)
1–5 years	984 (32.8)
5–10 years	677 (22.6)
> 10 years	572 (19.1)
Baseline intensity of neck pain (VAS)^b, d^	6.6 (2.2)
Baseline intensity of arm pain (VAS) ^b, e^	6.0 (2.5)
Baseline disability (NDI) ^b f^	37.2 (19.2)
Diagnostic procedures during the episode^a^	
X-Ray	699 (23.3)
MRI	602 (20.1)
CT scan	40 (1.3)
EMG	58 (1.9)
Other	135 (4.5)
Imaging findings^a^	
Disc degeneration	1335 (44.5)
Facet joint degeneration	230 (7.7)
Scoliosis	135 (4.5)
Spinal stenosis	63 (2.1)
Disc protrusion	270 (9.0)
Disc herniation (extrusion)	518 (17.3)
Other imaging findings	388 (12.9)
No findings	995 (33.2)
Clinical diagnosis	
Spinal stenosis	63 (2.1)
Disc herniation/protrusion	665 (22.2)
Nonspecific syndrome	2273 (75.7)
Treatments^a^	
Analgesics	1959 (65.3)
NSAIDs	1826 (60.8)
Steroids	190 (6.3)
Muscle relaxants	736 (24.5)
Opioids	52 (1.7)
Other	673 (22.4)
Non pharmacological treatments^a^	
Physical therapy	175 (5.8)
Rehabilitation	343 (11.4)
Neuro-reflexotherapy	2580 (86.0)
Surgery	8 (0.3)
Other treatments^a^	
Rhizolysis	3 (0.1)
EpiduraI injections	13 (0.4)
Referral to pain unit	12 (0.4)
Other treatments^g^	31 (1.0)

^a^ Frequency (%) ^b^ Mean (sd) ^c^ Median (p25;p75) ^d^:Patient with VAS for NP > 0 (*n* = 2961)^e^:Patient with VAS for AP > 0 (*n* = 2188)^f^:Patient with NDI > 0 (*n* = 1500)^g^:Includes; ozone injections, spinal manipulation, acupuncture, and homeopathy,

**Fig. 1:**
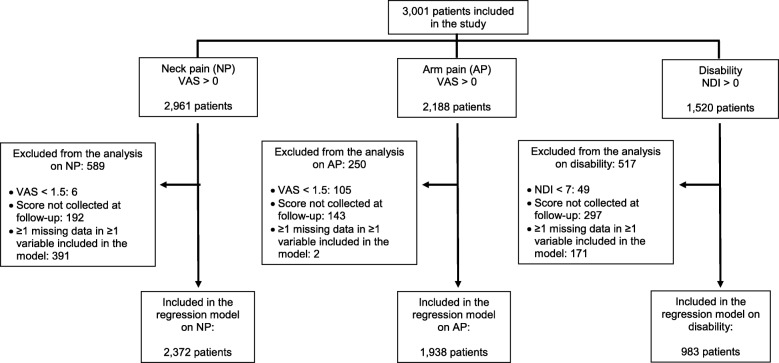
Flow chart showing the number of patients whose data were included in the regression models

At baseline, 2961 patients reported some degree of NP (i.e., VAS > 0), 2188 reported some degree of AP, and 1500 reported some degree of disability (i.e., NDI > 0). Data from 6 patients were excluded from the regression model on NP because their baseline scores were below the threshold required for any potential improvement to be “clinically relevant”. For the same reason, data from 105 and 49 patients were excluded from the regression models on AP and disability, respectively. All the reasons leading to the exclusion of data from the regression models, are shown in Fig. 1.

Among the patients included in the corresponding models, 1716 (72,3%) showed a clinically relevant improvement in NP at the 3 month assessment and the remaining 656 did not, while 507 patients (51,6%) showed a clinically relevant improvement in disability and 476 did not (Table 2). Among the 1938 patients who reported pain referred down to the arm (AP) when entering the study, 1371(70.7%) showed a clinically relevant improvement 3 months later, and 567 did not (Table 2).Conversely, AP appeared during the study period in 5 patients who had not reported it at baseline. Among these patients, median (p25;p75) VAS score for AP at the follow-up assessment was 3 (2;3).

**Table 2:** Characteristics of patients in whom neck pain, arm pain and disability improved and did not improve

Variables	Improvement of neckpain		Improvement of armpain		Improvement of disability	
	No (*n* = 656)	Yes (*n* = 1716)	No (*n* = 567)	Yes (*n* = 1371)	No (*n* = 476)	Yes (*n* = 507)
Gender (female)^a^	505 (77.0)	1263 (73.6)	435 (76.7)	1053 (76.8)	345 (72.5)	360 (71.0)
Age (years)^b^	48.6 (17.9)	50.9 (15.3)	50.5 (15.7)	51.6 (14.4)	47.8 (18.7)	48.3 (16.2)
Employment status^a^						
Not applicable	374 (57.0)	695 (40.5)	267 (47.1)	457 (33.3)	314 (66.0)	269 (53.1)
Not working	67 (10.2)	101 (5.9)	50 (8.8)	77 (5.6)	57 (12.0)	49 (9.7)
Working	215 (32.8)	920 (53.6)	183 (32.3)	664 (48.4)	105 (22.1)	189 (37.3)
Duration of the pain episode (days) ^c^	180 (60; 365)	180 (90; 365)	180 (90; 365)	240 (90; 365)	90 (32.5; 195)	120 (60; 365)
Duration of the pain episode categorized (days) ^a^						
Acute (< 90 days)	259 (39.5)	570 (33.2)	175 (30.9)	370 (27.0)	236 (49.6)	222 (43.8)
Chronic (≥90 days)	397 (60.5)	1146 (66.8)	392 (69.1)	1001 (73.0)	240 (50.4)	285 (56.2)
Time since first episode (years)^a^						
< 1	168 (25.6)	339 (19.8)	110 (19.4)	245 (17.9)	146 (30.7)	144 (28.4)
1–5	242 (36.9)	571 (33.3)	209 (36.9)	470 (34.3)	179 (37.6)	169 (33.3)
5–10	125 (19.1)	409 (23.8)	125 (22.1)	336 (24.5)	80 (16.8)	90 (17.8)
> 10	98 (14.9)	346 (20.2)	102 (18.0)	278 (20.3)	55 (11.6)	73 (14.4)
Baseline intensity of neck pain (VAS)^b^	6.0 (2.4)	6.7 (2.2)	6.6 (2.1)	7.1 (1.9)	5.6 (2.3)	6.1 (2.3)
Baseline intensity of arm pain (VAS)^b^	5.5 (2.5)	6.1 (2.5)	5.6 (2.4)	6.5 (2.2)	5.3 (2.5)	5.4 (2.5)
Baseline disability (NDI)^b^	34.1 (20.5)	37.0 (18.6)	39.5 (20.0)	41.8 (16.5)	32.8 (19.0)	38.4 (18.4)
Diagnostic procedures during the episode^a^						
X-Ray	122 (18.6)	457 (26.6)	118 (20.8)	348 (25.4)	80 (16.8)	101 (19.9)
MRI	115 (17.5)	364 (21.2)	109 (19.2)	285 (20.8)	65 (13.7)	110 (21.7)
CT scan	7 (1.1)	29 (1.7)	9 (1.6)	19 (1.4)	2 (0.4)	7 (1.4)
EMG	15 (2.3)	27 (1.6)	17 (3.0)	27 (2.0)	14 (2.9)	9 (1.8)
Other	36 (5.5)	71 (4.1)	39 (6.9)	60 (4.4)	29 (6.1)	19 (3.8)
Imaging findings^a^						
Disc degeneration	303 (46.2)	659 (38.4)	312 (55.0)	602 (43.9)	266 (55.9)	294 (58.0)
Facet joint degeneration	73 (11.1)	103 (6.0)	74 (13.1)	77 (5.6)	68 (14.3)	42 (8.3)
Scoliosis	25 (3.8)	75 (4.4)	24 (4.2)	61 (4.5)	21 (4.4)	28 (5.5)
Spinal stenosis	12 (1.8)	32 (1.9)	16 (2.8)	29 (2.1)	11 (2.3)	9 (1.8)
Disc protrusion	75 (11.4)	95 (5.5)	76 (13.4)	115 (8.4)	52 (10.9)	40 (7.9)
Disc herniation (extrusion)	112 (17.1)	234 (13.6)	123 (21.7)	245 (17.9)	79 (16.6)	117 (23.1)
Other findings	72 (11.0)	201 (11.7)	77 (13.6)	168 (12.3)	58 (12.2)	99 (19.5)
No findings	131 (20.0)	756 (44.1)	116 (20.5)	587 (42.8)	14 (2.9)	29 (5.7)
Clinical diagnosis						
Spinal stenosis	12 (1.8)	32 (1.9)	16 (2.8)	29 (2.1)	11 (2.3)	9 (1.8)
Disc protrusión/Herniation	164 (25.0)	285 (16.6)	173 (30.5)	302 (22.0)	117 (24.6)	143 (28.2)
Nonspecific syndrome	480 (73.2)	1399 (81.5)	378 (66.7)	1040 (75.9)	348 (73.1)	355 (70.0)
Treatments						
Analgesics	419 (63.9)	1139 (66.4)	391 (69.0)	959 (70.0)	339 (71.2)	353 (69.6)
NSAIDs	387 (59.0)	1062 (61.9)	352 (62.1)	888 (64.8)	310 (65.1)	347 (68.4)
Steroids	60 (9.2)	98 (5.7)			53 (11.1)	42 (8.3)
Muscle relaxants	170 (25.9)	438 (25.5)	147 (25.9)	347 (25.3)	142 (29.8)	170 (33.5)
Opioids	11 (1.7)	22 (1.3)	10 (1.8)	24 (1.8)	14 (2.9)	15 (3.0)
Other	145 (22.1)	362 (21.1)	143 (25.2)	290 (21.2)	148 (31.1)	182 (35.9)
Non pharmacological treatments						
Physical therapy	38 (5.8)	100 (5.8)	33 (5.8)	72 (5.3)	24 (5.0)	28 (5.5)
Rehabilitation	84 (12.8)	172 (10.0)	76 (13.4)	164 (12.0)	74 (15.6)	64 (12.6)
Neuro-reflexotherapy	428 (65.2)	1574 (91.7)	419 (73.9)	1332 (97.2)	252 (52.9)	379 (74.8)
Surgery	3 (0.5)	3 (0.2)	2 (0.4)	3 (0.2)	1 (0.2)	0 (0.0)
Other treatments						
Rhizolysis	1 (0.2)	2 (0.1)	1 (0.2)	2 (0.1)	2 (0.4)	1 (0.2)
Epidural injections	2 (0.3)	9 (0.5)	2 (0.4)	8 (0.6)	3 (0.6)	4 (0.8)
Referral to pain unit	7 (1.1)	4 (0.2)	7 (1.3)	4 (0.3)	9 (1.9)	2 (0.4)
Othertreatments^d^	3 (0.5)	17 (1.0)	5 (0.9)	11 (0.8)	4 (0.8)	7 (1.4)

^a^Frequency (%). ^b^ Mean (sd). ^c^ Median (p25;p75). ^d^ Includes; injections of ozone, spinal manipulation, acupuncture, and homeopathy

The multivariable logistic regression model showed that the factors which, at baseline, predicted a clinically relevant improvement in NP 3 months later, were, from highest to lowest frequency in bootstrapping validation (Table 3a): being treated with neuro-reflexotherapy, pain being acute (vs. chronic), arm pain being less severe, working (vs. “receiving compensation assistance for NP” or “non-worker”), not showing signs of disc degeneration on imaging, suffering from “non-specific” pain (vs. pain caused by spinal stenosis or disc herniation/protrusion), being female, and suffering higher baseline intensity of neck pain. The assessment of the calibration of the model showed that the frequency of expected and observed probabilities of improvement were similar (Hosmer-Lemeshow test, *p* = 0.383). Discrimination of the model was good, with an AUC of 0.718. Overfitting was 0.020.

**Table 3:** Predictive models for improvement

a. Predictive model for the improvement of neck pain (*n* = 2372).^a,b^			
Variables	Odds ratio	p	Frequency in bootstrapping validation^c^ (100)
	(I.C. 95%)		
Being treated with neuro-reflexotherapy	9.90 (6.81; 14.38)	< 0.001	100
Pain being chronic (≥ 90 days)	0.53 (0.41; 0.70)	< 0.001	98
Baseline intensity of arm pain (VAS)^d^	0.93 (0.90; 0.97)	< 0.001	95
Employment status (ref. working)			
Non worker	0.87 (0.69; 1.10)	0.259	94
Receiving financial compensation for neck pain	0.48 (0.33; 0.69)	< 0.001	94
Signs of disc degeneration on imaging	0.77 (0.62; 0.95)	0.017	68
Clinical diagnosis (ref. nonspecific pain)			
Spinal stenosis	0.78 (0.39; 1.56)	0.482	63
Disc herniation/protusion	0.63 (0.49; 0.81)	< 0.001	63
Female	0.77 (0.61; 0.97)	0.030	59
Baseline intensity of neck pain (VAS)^d^	1.06 (1.00; 1.13)	0.041	38
Constant	0.84 (0.60; 1.18)	0.325	–
b. Predictive model for the improvement of pain referred down into the arm (*n* = 1938).^e,f^			
Variables	Odds ratio	p	Frequency in bootstrapping validation^g^ (100)
	(I.C. 95%)		
Being treated with neuro-reflexotherapy	16.96 (10.90; 26.37)	< 0.001	100
Baseline intensity of arm pain (VAS) ^h^	1.17 (1.10; 1.24)	< 0.001	96
Pain being chronic (≥ 90 days)	0.57 (0.43; 0.75)	< 0.001	84
Signs of disc degeneration on imaging	0.68 (0.54; 0.85)	0.001	83
Baseline intensity of neck pain (VAS)^d^	0.91 (0.85; 0.98)	0.010	73
Clinical diagnosis (ref. nonspecific pain)			
Spinal stenosis	0.57 (0.52; 0.86)	0.088	56
Disc herniation/protrusion	0.67 (0.52; 0.86)	0.002	56
Constant	0.31 (0.19; 1.24)	< 0.001	–
c. Predictive model for the improvement for disability (*n* = 983).^i,j^			
Variables	Odds ratio	P	Frequency in bootstrapping validation^k^ (100)
	(I.C. 95%)		
Baseline intensity of arm pain (VAS)^l^	0.89 (0.85; 0.93)	< 0.001	99
Being treated with neuro-reflexotherapy	2.92 (1.90; 4.49)	< 0.001	97
Employment status (ref. working)			
Non worker	0.69 (0.49; 0.97)	0.031	90
Receiving financial compensation for neck pain	0.45 (0.28; 0.73)	0.001	90
Baseline disability (NDI)^m^	1.02 (1.01; 1.02)	0.002	84
Signs of facet joint degeneration on imaging	0.60 (0.39; 0.93)	0.023	73
Pain being chronic (≥ 90 days)	0.65 (0.46; 0.91)	0.012	56
Constant	0.89 (0.59; 1.34)	0.589	–

^a^The number of patients who reported some degree of pain referred down to the arm (AP) at baseline (VAS > 0), was 2961,6 had baseline scores below the cut-off for considering potential improvements as “clinically relevant”, 583 had missing data at the baseline or the follow-up assessment, and 2372 were included in the model

^b^AUC = 0.718 (95%CI; 0.694–0.742). Hosmer-Lemeshow: chi^2^ = 0.383

^c^Overfitting = 0.020. Shrinkage factor = 0.906

^d^VAS: Visual Analog Scale (range from better to worse; 0–10)

^e^The number of patients who reported some degree of neck pain (VAS > 0) at baseline, was 2961, 18 had baseline scores below the cut-off for considering potential improvements as “clinically relevant”, 238 had missing data at the baseline or the follow-up assessment, and 2372 were included in the model

^f^AUC = 0.717 (95%CI; 0.691–0.742). Hosmer-Lemeshow: chi^2^ = 0.369

^g^Overfitting = 0.030. Shrinkage factor = 0.882

^h^VAS: Visual Analog Scale (range from better to worse; 0–10)

^i^The number of patients who reported some degree of disability at baseline (NDI > 0), was 1500,49 had baseline scores below the cut-off for considering potential improvements as “clinically relevant”, 468 had missing data at the baseline or the follow-up assessment, and 983 were included in the model

^j^AUC = 0.677 (95%CI; 0.644–0.711). Hosmer-Lemeshow: chi^2^ = 0.128

^k^Overfitting = 0.037. Shrinkage factor = 0.787

^l^VAS: Visual Analog Scale (range from better to worse; 0–10)

^m^Score on the Neck Disability Index (range from better to worse, 0–100)

Factors predicting a clinically relevant improvement in AP 3 months later, were: being treated with neuro-reflexotherapy, arm pain being more severe, pain being acute (vs. chronic), not showing signs of disc degeneration on imaging, neck pain being less severe, and suffering from “non-specific” pain (vs. pain caused by spinal stenosis or disc herniation/protrusion) (Table 3b). The assessment of the calibration of the model showed that the frequency of expected and observed probabilities of improvement were similar (Hosmer-Lemeshow test, *p* = 0.369). Discrimination of the model was good, with an AUC of 0.717. Overfitting was 0.030.

Factors predicting a clinically relevant improvement in disability 3 months later, were: arm pain being less severe, being treated with neuro-reflexotherapy, working (vs. “receiving financial compensation for NP” or “non worker”), baseline disability being higher, not showing signs of facet joint degeneration on imaging, and pain being acute (vs. chronic) (Table 3c). The assessment of the calibration of the model showed that the frequency of expected and observed probabilities of improvement were similar (Hosmer-Lemeshow test, *p* = 0.480). Discrimination of the model was poor, with an AUC of 0.664. Overfitting was 0.037.

**Fig. 2:**
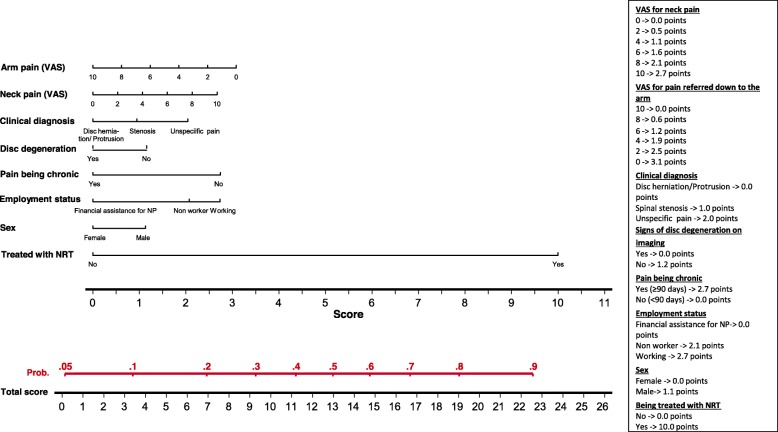
Nomogram for improvement of neck pain

**Fig. 3:**
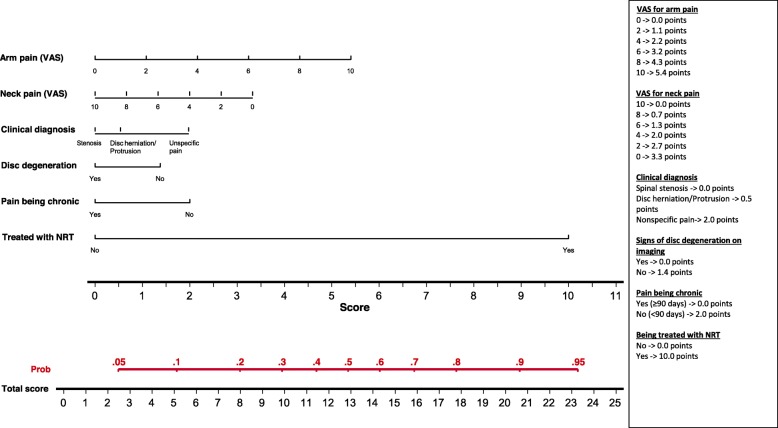
Nomogram for improvement of arm pain

**Fig. 4:**
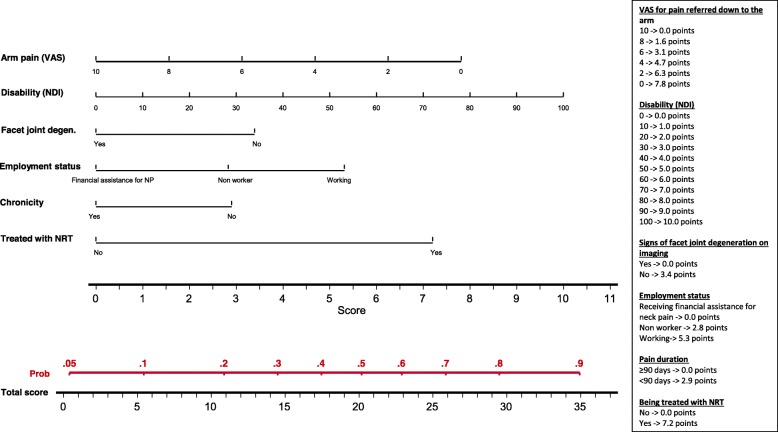
Nomogram for improvement of disability

Figures 2, 3 and 4 show the nomograms corresponding to the models on improvement of NP, AP and disability.

## Discussion

Results from this study show that it is feasible to implement a registry for NP patients treated in routine clinical practice. The registry included all patients seeking care for NP in the participating centers, where they were treated following standard practice in routine clinical practice. Clinically relevant data were gathered using previously validated methods [14, 16, 18, 24–26], and the proportion of losses to follow-up was below 7%. Data from this registry make it possible to develop predictive models which are valid to determine the likelihood of improvement, factoring in patients’ characteristics and clinical decisions, and nomograms to facilitate the use of these models in routine practice (Table 3, Figs. 2-4).

However, a registry analysis is an observational study, and “association” does not necessarily imply “causality”. Therefore, results showing the association between a given variable and a better or worse prognosis should be interpreted cautiously, taking clinical plausibility into account. For instance, the association between staying at work and improvement in NP, which is consistent with results from previous studies on NP [32, 33], might be due to the fact that staying active improves the evolution of NP, and/or to the fact that subjects staying at work are those with a mental and personal attitude that are conducive to a better prognosis.

Nevertheless, results from this study are in line with those from previous studies and are clinically plausible; most imaging findings are unlikely to predict the clinical evolution of NP, AP or disability, unless there is a correlation between clinical and radiological findings [34]; radicular pain caused by symptomatic spinal stenosis or disc herniation is harder to treat than referred pain associated with common NP; irrespective of the treatments applied and other patient characteristics, chronic (vs. acute) pain is associated with a worse prognosis for pain and disability; neuro-reflexotherapy is associated with a significant improvement in pain and disability [16, 24, 27]; staying at work is associated with a better prognosis [32, 33]; and a higher baseline value for a given variable (NP, AP or disability) leaves more room for its improvement, whereas the prognosis is worse for patients who are more severely affected (in terms of the other variables).

It is likely that the evolution of disability is affected by additional variables which were not assessed in this registry. In fact, discrimination was good in the models on NP and AP, whereas it was poor in the model on disability. Additionally, baseline mean score for disability was mild, and in a significant number of patients it was below the value of the minimal clinically important change. As a result, the model on disability only included data from approximately 40% of the patients. Despite all of the above, for all models discrimination was > 0.663 and calibration was acceptable, which suggests that nomograms may be applicable in clinical practice for early identification of patients who are at a higher risk of becoming chronic.

Many different treatment modalities are used for the treatment of NP, but very few are supported by solid evidence on efficacy or effectiveness deriving from high quality randomized controlled trials (RCTs) [3, 16, 35–49]. In fact, few RCTs on spinal pain have focused on NP [3], maybe because it is less prevalent than low back pain [1, 2]. As a result, clinical practice in the NP field is largely driven by the results from RCTs on other pain conditions, mainly low back pain, and RCTs to assess the efficacy, effectiveness and cost-effectiveness of treatments for NP are suitable [3].

Registries cannot substitute RCTs, and are not valid for proving the efficacy or effectiveness of treatments. However, they can be useful to assess the prognostic value of procedures which have previously shown to be efficacious through appropriate randomized clinical trials, or are assumed to be effective based on the evidence on their effect in other types of spinal pain. None of the pharmacological groups which were analyzed in this study was associated with clinical improvement. This might be due to the fact that, for the treatment of patients with NP, drugs are essentially designed to offer symptomatic pain relief, while this study focused on the improvement of the pain episode at 3 months. For the same reason, the timeframe of this study may be inappropriate to capture the effect of treatments aiming to prevent relapse or requiring longer periods to have an effect, such as exercise or surgery.

Previous studies and systematic reviews on prognosis factors for neck pain vary substantially in design and setting, and methodological shortcomings have led to uncertainty about the reliability of the prognostic factors which have been suggested [4]. In fact, many original studies on prognostic factors are re-analyses of randomized controlled trials with relatively small samples, which aim to identify subsets of patients who respond better to some form of specific treatment [4]. This makes it inappropriate to compare their results with those from this study, in which a large population recruited in routine clinical practice and receiving a broad array of treatments, was analyzed.

The representativeness of the sample does not appear to be a major concern; recruiting centers were spread over 11 out of the 17 administrative regions existing in Spain, they included state-owned (i.e., “public”) and private practices (both working for the Spanish National Health Service and for private institutions, both for profit and non for profit), and they included primary care, physical therapy and specialty centers. All patients seeking care in these practices were screened consecutively, all those who complied with inclusion criteria were included, none were excluded, losses to follow-up were below 7%, and patients’ characteristics are in line with the typical patient requesting care for neck pain (Table 1). However, since most spinal neck surgery in Spain is performed at Neurosurgery Departments, and none participated in this study, only 8 patients (0.3%) underwent surgery, which makes this study unsuitable to assess the potential predictive value of spinal neck surgery on the evolution of a pain episode.

In fact, this study and the registry from which it derives, have a number of limitations. At the design phase of this study, it was decided that the sample should represent patients seeking health care for neck pain, reflecting prevalent (and not only incidental) cases. As a result, the sample included both patients with acute pain and those with (exacerbations of) chronic pain. The proportion of acute and chronic patients might have been different if the proportion of primary and specialty centers had been different.

Should the study have been conducted in a different setting, the proportion of patients receiving each form of treatment might also have been different. In fact, this study did not gather any data on the rationale for prescribing a specific treatment to a given patient. Therefore, such a rationale and how strictly the clinicians involved in this study followed indication criteria for each treatment, are unknown. However, this is usual in routine practice, and this study did not aim to assess the therapeutic effect of any treatments in experimental conditions, but their prognostic value in routine practice. Moreover, this study included a broad array of patients, recruited in both primary and specialty care centers, and the analysis made it possible to assess the association between each one of their characteristics and their prognosis for pain and disability. Future studies should explore the generalizability of these models to other cultural contexts.

In Spain, especially within the Government run health system (i.e., the National Health Service), patients are first seen by a primary care physician, who acts as a “gatekeeper”, checks for the presence of “red flags” and refers patients with indication criteria for a specialized treatment to the appropriate specialist. Therefore, some treatments are almost exclusively applied in Services specialized in that given treatment (e.g., physical therapy, neuroreflexotherapy, rehabilitation or orthopedic surgery). This implies that, in this study, the prognostic value of being treated in one or other Service could not be explored because multicolinearity was detected between “treatment received” and “specialty”. The prognostic value of the type of clinician who treats the patient should be explored in further studies, especially if conducted in settings where patients with neck pain are the main decision makers when deciding on the type of clinician they chose to seek care from.

Losses to follow up beyond 3 months in this register are unknown and should be assessed in further studies. This study focused on the clinical prognosis of a single NP episode, and analyzing factors associated with relapses was not within its scope and would require longer follow-up periods.

The health care centers invited to join this study were selected because of their previous involvement in research on spinal pain, and are not a random sample of health care centers. As a result, no neurosurgery practice was included in the sample, and participating clinicians may be more evidence-based in their practice than average, or more positively predisposed than average for implementing a registry on NP. Nevertheless, these centers represented a feasible and adequate platform to test run this registry in the context of routine practice, although It may be more difficult to implement registries in environments where the clinical community is less accustomed to e-health.

This registry did not include any psychological variables. This is due to the fact that, to date, the only psychological variables potentially influencing the prognosis of spinal pain which have been assessed in the Spanish cultural setting are fear avoidance beliefs and catastrophizing, and their influence has shown to be clinically irrelevant or null [20, 21, 50]. Nonetheless, pain is known to be a sensory and emotional experience which may be affected by psychological factors [51]. Therefore, psychological variables should be added to this registry as soon as future research identifies those with a prognostic value on neck pain in this setting.

## Conclusions

Results from this study suggest that the probability that a patient with neck pain will experience improvements in neck pain, arm pain and disability within a 3 months period, can be quantified using the following data; baseline scores for neck pain, arm pain and disability, whether neck pain is acute or chronic, whether it is “nonspecific” (vs. caused by disc herniation or spinal stenosis), whether the patient is male or female, whether he/she is treated with neuro-reflexotherapy, whether he/she stays at work (vs. does not work or receives financial compensation for neck pain), and whether he/she shows some specific images on MRI.

## Data Availability

The datasets analyzed during the current study are available from the corresponding author on reasonable request.

## Abbreviations

AP: Pain referred down to the arm
AUC: Area under the ROC curve
EMG: Electromyography
MCIC: Minimal clinically important change
MRI: Magnetic resonance imaging
NDI: Neck disability index
NP: Neck pain
NSAIDs: Nonsteroidal anti-inflammatory drugs
RCT: Randomized controlled trial
ROC: Receiver operating characteristic
SD: Standard deviation
SNHS: Spanish National Health Service
VAS: Visual analog scale
